# Using controlled attenuation parameter combined with ultrasound to survey non-alcoholic fatty liver disease in hemodialysis patients: A prospective cohort study

**DOI:** 10.1371/journal.pone.0176027

**Published:** 2017-04-20

**Authors:** Yi-Hao Yen, Jin-Bor Chen, Ben-Chung Cheng, Jung-Fu Chen, Kuo-Chin Chang, Po-Lin Tseng, Cheng-Kun Wu, Ming-Chao Tsai, Ming-Tsung Lin, Tsung-Hui Hu

**Affiliations:** 1 Division of Hepato-Gastroenterology, Department of Internal Medicine, Kaohsiung Chang Gung Memorial Hospital and Chang Gung University College of Medicine, Kaohsiung, Taiwan; 2 Division of Nephrology, Department of Internal Medicine, Kaohsiung Chang Gung Memorial Hospital and Chang Gung University College of Medicine, Kaohsiung, Taiwan; 3 Division of Endocrinology & Metabolism, Department of Internal Medicine, Kaohsiung Chang Gung Memorial Hospital and Chang Gung University College of Medicine, Kaohsiung, Taiwan; Taipei Veterans General Hospital, TAIWAN

## Abstract

**Background and aims:**

Controlled attenuation parameter (CAP) is a non-invasive method for measuring hepatic steatosis (HS). Non-alcoholic fatty liver disease (NAFLD) is closely related to cardiovascular diseases (CVDs). CVDs are the leading cause of morbidity and mortality in hemodialysis patients. The aim of this study was to investigate the prevalence of NAFLD in hemodialysis patients.

**Method:**

We prospectively enrolled patients undergoing chronic hemodialysis, as well as patients with normal renal function who served as controls. The control group patients were referred by an endocrinologist to be tested for NAFLD; most of these patients had diabetes, hypertension, or dyslipidemia. We excluded those with excess alcohol intake, use of drugs known to induce HS, chronic viral hepatitis, or CAP failure. CAP ≥ 238 dB/m was used as a cutoff suggesting HS. An increased liver kidney contrast, as defined by ultrasound, was used to make the diagnosis of HS.

**Results:**

Three hundred and forty-three hemodialysis patients and 252 control group patients were enrolled. Among the hemodialysis patients, 192 (56.0%) had CAP- or ultrasound-identified HS compared with 91 (26.5%) who only had ultrasound-identified HS (P<0.001). Among the control group patients, 212 (84.1%) had CAP- or ultrasound-identified HS compared with 180 (71.4%) who only had ultrasound-identified HS (P<0.001).

**Conclusions:**

The prevalence of NAFLD in the hemodialysis patients was 56%. The number of diagnoses of NAFLD made by using CAP combined with ultrasound was more than 2 times the number made with ultrasound alone in the hemodialysis patients. Therefore, we suggest the use of CAP combined with ultrasound to screen for NAFLD in hemodialysis patients.

## Introduction

It is well known that cardiovascular diseases (CVDs) are the leading cause of morbidity and mortality in chronic kidney disease patients [1–6]. In patients who undergo dialysis treatment, there are well established risk factors that contribute to the development of CVDs, including hypertension, diabetes, dyslipidemia, and being overweight [7].

Non-alcoholic fatty liver disease (NAFLD) is strongly associated with obesity, insulin resistance, hypertension, and dyslipidemia, and is now regarded as the liver manifestation of metabolic syndrome [8–10], which is a highly atherogenic condition, even at a very early age [11–13]. Natural history studies have reported that the increased age-related mortality observed in patients with NAFLD is attributable to cardiovascular as well as liver-related deaths [11,14].

According to these observations, hemodialysis patients who develop NAFLD because of shared etiological factors (such as the components of metabolic syndrome) probably have a much faster progression of the atherosclerotic process and development of adverse CVD events than patients without NAFLD [15]. That said, the prevalence of NAFLD in patients undergoing dialysis remains unclear. However, the main risk factors responsible for the development of NAFLD are commonly observed in dialysis patients. Therefore, it is logical to expect that end-stage renal disease (ESRD) patients on maintained hemodialysis would also have a high prevalence of NAFLD.

The controlled attenuation parameter (CAP) is a technology that is used to measure the degree of ultrasound (US) attenuation by hepatic fat at the central frequency of the FibroScan (Echosens, Paris, France) [16]. A FibroScan measurement is also less likely to be influenced by sampling error than a liver biopsy because it is made by exploring a liver volume approximately 100 times larger than that explored using a liver biopsy. Moreover, a meta-analysis showed that the CAP has good sensitivity and specificity for detecting hepatic steatosis (HS) [17].

US is the most commonly used imaging tool for detecting HS. In a previous study, US was found to have a 92% sensitivity and 100% specificity for the detection of HS compared to the use of biopsy as the standard method [18]. Another study also demonstrated that US has both high sensitivity and specificity for evaluating HS when the fat content is over 20% [19].

Thus far, there have only been limited reports regarding the use of CAP to evaluate HS in hemodialysis patients [20–23]. The aim of this study, therefore, was to investigate the prevalence of NAFLD in hemodialysis patients using CAP and US. Furthermore, we also investigated the clinical factors associated with HS in hemodialysis patients to determine if they are different from those in patients with normal renal function.

## Methods

### Subjects

This was a prospective cohort study. From July 2014 to September 2014, all consecutive patients treated with chronic hemodialysis for at least 6 months in Kaohsiung Chang Gung Memorial Hospital, Taiwan, were included in the study. During the same period of time, we enrolled patients with normal renal function to serve as controls. Normal renal function was defined by normal serum creatinine levels for each gender, with levels of 0.64~1.27 mg/dL considered normal for male patients and levels of 0.44~1.03 mg/dL considered normal for female patients (these are the definitions used in our hospital). The control group patients were referred by an endocrinologist to be tested for NAFLD; most of these patients had diabetes mellitus (DM), hypertension, or dyslipidemia. The exclusion criteria for both the hemodialysis group and the control group were as follows: Subjects with positive hepatitis B surface antigen or antibody against hepatitis C virus, use of drugs known to induce HS, excessive alcohol consumption (>21 drinks per week in men and >14 drinks per week in women over the preceding 2-year period) [24], or CAP failure. Three hundred and forty-three hemodialysis patients and 252 control group patients were enrolled. Written informed consent was obtained from each of the participants in the study. The study protocol adhered to the ethical guidelines of the 1975 Declaration of Helsinki and was approved by the ethics committee of Chang Gung Memorial Hospital (IRB no. 101-1907B).

### Clinical assessment

The assessment included an interview conducted by a nurse. The medical history, current use of prescribed drugs, smoking status, and alcohol consumption of each patient were recorded using standard questionnaires. The body mass index (BMI) of each patient was calculated by dividing the patient’s body weight (in kg) by his or her body height (in m2). After each patient fasted for 8 hours overnight, his or her blood was sampled for assays of fasting lipids, glucose, and renal and liver function.

### Controlled attenuation parameter measurement

Liver stiffness measurement (LSM) and CAP measurements were performed by two experienced operators using the FibroScan (Echosens, Paris, France). All patients were measured using the 3.5 MHz standard M+ probe. The principles of CAP have been described elsewhere [16]. The final CAP value as the median of the individual CAP values and was expressed in dB/m. The ranges for different grades of steatosis were a CAP value of 238 dB/m to 259 dB/m for steatosis grade 1 (S1), a CAP value of 260 dB/m to 291 dB/m for S2, and a CAP value of 292 dB/m or above for S3 [16].

### US examination

Predefined criteria were used to determine the severity of HS. These included the presence of increased hepatorenal contrast for mild HS (i.e., S1); the presence of both bright echoes and increased hepatorenal contrast, as well as vessel blurring, for moderate HS (i.e., S2); and the presence of posterior beam attenuation and non-visualization of the diaphragm, in addition to the criteria for moderate HS, for severe HS (i.e., S3). The liver image was assessed to be normal if the texture was homogenous, fine level echoes were seen, the area was isoechoic compared to the renal cortex, and the hepatic vessels and diaphragm were adequately visualized [19].

### Statistical analysis

Baseline characteristics and clinical variables were summarized as mean ± standard deviation, median (25th-75th percentile), or percentage. The chi-square test and independent two-sample t-test were used, as appropriate, to assess the significance of any differences in the distributions. The Spearman correlation coefficient was used to investigate the correlation between the CAP values and the steatosis grades assessed by US. A multivariate logistical regression analysis was conducted to identify any patient characteristics independently associated with CAP- or US-identified HS. A univariate analysis was first performed on each of the considered independent variables to select candidate variables for the multivariate analysis. A *P* value of less than 0.05 was considered significant. Statistical analyses were performed using Stata software version 11.0.

## Results

### Demographic characteristics of the analyzed patients

Table 1 provides a description of the demographic characteristics of the analyzed patients. Of the 343 hemodialysis patients, 45.2% were male, the mean age was 63.2 years (range: 20.1 to 89.0 years), and 30.6% of the patients had DM. Furthermore, the median CAP value for these patients was 234 dB/m. Of the 252 control group patients, 50.8% were male, the mean age was 57.8 years (range: 21.5 to 93.3 years), and 38.9% had DM, while the median CAP value was 276 dB/m. The hemodialysis patients were significantly older than the control group patients, while the BMI, alanine aminotransferase (ALT), aspartate aminotransferase (AST), total bilirubin, albumin, cholesterol, low-density lipoprotein (LDL), and CAP values were significantly lower among the hemodialysis patients. In addition, the fasting sugar levels, the LSM values, the proportions of male patients with high-density lipoprotein (HDL)< 40 mg/dL and female patients with HDL< 50 mg/dL, and the proportion of patients with triglyceride >150 mg/dL were significantly higher among the hemodialysis patients (P < 0.05).

**Table 1:** Clinical characteristics of patients.

Variables	Hemodialysis (N = 343)	Control (N = 252)	*P* value
Age(yr)	63.2±12.3	57.8±11.8	<0.001
Male	155 (45.2%)	128 (50.8%)	0.18
BMI (kg/m^2^)			<0.001
<24	246 (72.1%)	73 (30.3%)	
24–30	87 (25.5%)	129 (53.5%)	
>30	8 (2.4%)	39 (16.2%)	
DM (%)	105 (30.6%)	98 (38.9%)	0.04
AST (IU/L)	19.8±8.9	35.9±23.0	<0.001
ALT (IU/L)	16.5±12.2	41.8±30.6	<0.001
Bilirubin (mg/dL)	0.4±0.6	1.0±1.0	<0.001
Albumin (g/dL)	3.9±0.3	4.6±0.4	<0.001
Fasting sugar (mg/dL)	147.9±70.5	113.0±31.5	<0.001
HDL, male< 40, female < 50(mg/dL)	171 (51.2%)	38 (17.8%)	<0.001
LDL (mg/dL)	87.5±30.9	99.7±32.2	<0.001
Total cholesterol (mg/dL)	163.1±39.5	182.4±37.2	<0.001
VLDL(mg/dL)	29.0±17.3	24.9±12.4	0.004
Triglyceride >150 (mg/dL)	126 (37.2%)	56 (26.4%)	0.009
Platelet<150 (1000/μL)	93 (27.4%)	21 (20.4%)	0.16
LSM (kPa)	6.2 (4.7–8.4)	5.4 (4.4–8.5)	0.004
CAP (dB/m)	234 (201–268)	276 (239–311)	<0.001
Serum iron (mg/dL)	69.4±30.1	Not available	-
TIBC(mg/dL)	207.0±37.7	Not available	-
Ferritin (ng/mL)	432.2 (287–617.1)	Not available	-
Hemoglobin (g/dL)	10.5±1.2	Not available	-
Ultrasound steatosis grade			<0.001
S1	55 (16.0%)	61 (24.2%)	
S2	32 (9.3%)	105 (41.7%)	
S3	4 (1.2%)	14 (5.6%)	
CAP			<0.001
S1	63 (18.4%)	38 (15.1%)	
S2	53 (15.5%)	59 (23.4%)	
S3	47 (13.7%)	95 (37.7%)	

Data are shown as mean±SD, but LSM, and CAP are shown as median (interquantile). BMI, body mass index; DM, diabetes mellitus; ALT, alanine aminotransferase; AST, aspartate aminotransferase; HDL, High-density lipoprotein; CAP, Controlled Attenuation Parameter; LSM, liver stiffness measurement; LDL, low-density lipoprotein; VLDL, Very-low-density lipoprotein; TIBC, total iron-binding capacity

### Factors associated with CAP-identified HS

The multivariate analyses showed that high BMI (OR: 1.28, 95% CI: 1.16–1.40, P<0.001), low HDL (male< 40, female < 50 mg/dL) (OR: 2.15, 95% CI: 1.26–3.65, P = 0.005), and triglyceride >150 mg/dL (OR: 3.28, 95% CI: 1.88–5.73, P<0.001) were independent factors associated with CAP ≥238 dB/m in the hemodialysis patients. Among the control group patients, high BMI (OR:1.30, 95% CI: 1.12–1.51, P = 0.001) and high ALT (OR:1.03, 95%CI: 1.002–1.05, P = 0.032) were independent factors associated with CAP ≥238 dB/m (Table 2).

**Table 2:** Factors associated with CAP ≥ 238 dB/m vs. CAP <238 dB/m (logistic regression).

Variables	Hemodialysis				Control			
	Univariate		Multivariate		Univariate		Multivariate	
	OR (95% CI)	*P*	OR	*P*	OR (95% CI)	*P*	OR	*P*
Age	1.01 (0.99–1.02)	0.530	-		1.00 (0.98–1.03)	0.950	-	
Male	0.92 (0.60–1.42)	0.720	-		1.04 (0.58–1.86)	0.888	-	
BMI (kg/m^2^)	1.37 (1.26–1.49)	<0.001	1.28 (1.16–1.40)	<0.001	1.36 (1.22–1.52)	<0.001	1.30 (1.12–1.51)	0.001
DM	2.07 (1.30–3.30)	0.002	1.51 (0.81–2.82)	0.190	2.28 (1.19–4.38)	0.013	2.18 (0.82–5.79)	0.119
AST (IU/L)	0.99 (0.96–1.01)	0.260	-		1.02 (0.99–1.04)	0.151	-	
ALT(IU/L)	0.99 (0.98–1.01)	0.710	-		1.02 (1.00–1.04)	0.028	1.03 (1.002–1.05)	0.032
Fasting sugar (mg/dL)	1.01 (1.00–1.01)	0.001	0.99 (0.99–1.01)	0.940	1.01 (0.99–1.03)	0.264	-	
HDL, male< 40, female < 50(mg/dL)	4.20 (2.65–6.63)	<0.001	2.15 (1.26–3.65)	0.005	2.52 (0.84–7.52)	0.098	-	
LDL(mg/dL)	1.00 (0.99–1.01)	0.480	-		1.00 (0.99–1.01)	0.911	-	
Total cholesterol(mg/dL)	1.01 (0.99–1.01)	0.090	-		1.00 (0.99–1.01)	0.816	-	
Triglyceride >150(mg/dL)	5.88 (3.60–9.60)	<0.001	3.28 (1.88–5.73)	<0.001	4.48 (1.52–13.18)	0.006	5.22 (0.64–42.7)	0.124
Platelet(1000/μL)	0.73 (0.45–1.18)	0.200	-		0.52 (0.19–1.45)	0.212	-	
LSM (kPa)	1.00 (0.95–1.06)	0.890	-		1.01 (0.98–1.04)	0.671	-	

BMI, body mass index; DM, diabetes mellitus; ALT, alanine aminotransferase; AST, aspartate aminotransferase; HDL, High-density lipoprotein; CAP, Controlled Attenuation Parameter; LSM, liver stiffness measurement; LDL, low-density lipoprotein

### Factors associated with US-identified HS

The multivariate analyses showed that high BMI (OR: 1.21, 95% CI: 1.11–1.32, P<0.001) was an independent factor associated with US-identified HS in the hemodialysis patients. In addition, high BMI (OR:1.15, 95%CI: 10.2–1.29, P = 0.027) and low LSM (OR: 0.94, 95%CI: 0.88–0.99, P = 0.027) were independent factors associated with US-identified HS in the control group patients (Table 3).

**Table 3:** Factors associated with US-identified HS (logistic regression).

Variables	Hemodialysis				Control			
	Univariate		Multivariate		Univariate		Multivariate	
	OR (95% CI)	*P*	OR	*P*	OR (95% CI)	*P*	OR	*P*
Age	1.01 (0.99–1.03)	0.370	-		1.00 (0.98–1.03)	0.718	-	
Male	0.99 (0.61–1.61)	0.980	-		0.71 (0.41–1.23)	0.218	-	
BMI (kg/m^2^)	1.25 (1.16–1.36)	<0.001	1.21 (1.11–1.32)	<0.001	1.20 (1.10–1.31)	<0.001	1.15 (1.02–1.29)	0.027
DM	1.52 (0.92–2.52)	0.100	-		3.25 (1.71–6.17)	<0.001	2.67 (0.99–7.17)	0.052
AST (IU/L)	1.00 (0.97–1.03)	0.990	-		1.00 (0.98–1.01)	0.870	-	
ALT(IU/L)	1.01 (0.99–1.03)	0.180	-		1.02 (1.00–1.03)	0.026	1.02 (0.99–1.04)	0.061
Fasting sugar (mg/dL)	1.01 (1.002–1.01)	0.002	1.00 (0.99–1.01)	0.22	1.01 (0.99–1.03)	0.259	-	
HDL, male< 40, female < 50(mg/dL)	2.87 (1.71–4.81)	<0.001	1.59 (0.88–2.86)	0.12	1.42 (0.61–3.31)	0.420	-	
LDL(mg/dL)	0.99 (0.99–1.01)	0.850	-		1.00 (0.99–1.01)	0.846	-	
Total cholesterol(mg/dL)	1.00 (0.99–1.01)	0.310	-		1.00 (0.99–1.01)	0.478	-	
Triglyceride >150(mg/dL)	3.24 (1.97–5.33)	<0.001	1.74 (0.98–3.08)	0.06	2.25 (1.02–4.97)	0.044	1.12 (0.37–3.42)	0.839
Platelet(1000/μL)	0.95 (0.55–1.64)	0.870	-		0.17 (0.06–0.48)	0.001	0.25 (0.06–0.99)	0.050
LSM (kPa)	0.95 (0.89–1.02)	0.180	-		0.93 (0.90–0.97)	<0.001	0.94 (0.88–0.99)	0.027

BMI, body mass index; DM, diabetes mellitus; ALT, alanine aminotransferase; AST, aspartate aminotransferase; HDL, High-density lipoprotein; CAP, Controlled Attenuation Parameter; LSM, liver stiffness measurement; LDL, low-density lipoprotein

### Comparison of the diagnostic value of CAP combined with US versus US alone

Among the 343 hemodialysis patients, 192 (56.0%) had CAP- or US-identified HS compared with 91 (26.5%) who only had US-identified HS (P<0.001). Among the 252 control group patients, 212 (84.1%) had CAP- or US-identified HS compared with 180 (71.4%) who only had US-identified HS (P<0.001).

### The agreement between CAP and US-identified HS

**Fig 1:**
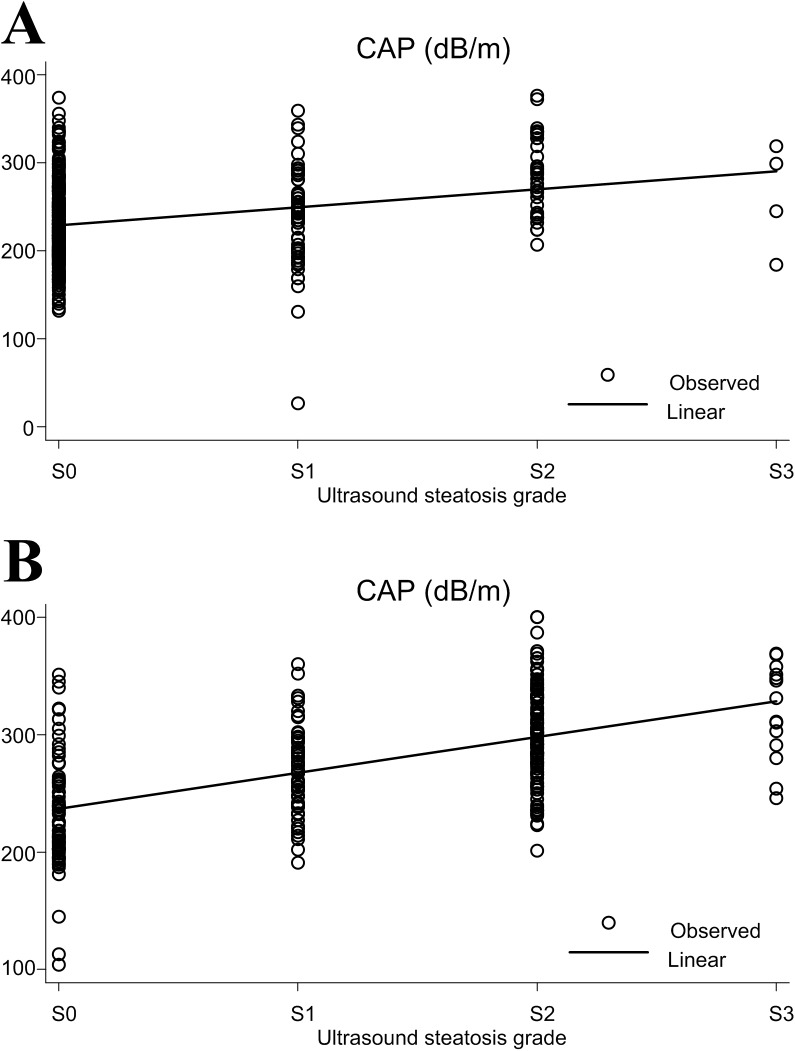
**The correlation between the controlled attenuation parameter (CAP) values and steatosis grades assessed by ultrasound in hemodialysis (A) and control group (B) patinets**.

The CAP values were significantly correlated with the steatosis grades identified by US for both the hemodialysis (Fig 1A) (ρ = 0.26, P<0.001) and control group patients (Fig 1B) (ρ = 0.51, P<0.001). The kappa value is 0.20 in the hemodialysis patients and 0.33 in the control group patients. The percentage of overall discordant cases is 48.98% in the hemodialysis patients and 64.29% in the control group patients.

### Comparison of NAFLD patients who had CAP combined with US-identified HS and NAFLD patients who had HS identified by CAP alone

The average BMI level, fasting sugar level, and the proportion of patients with low HDL levels and high triglyceride levels were higher in NAFLD patients who had CAP combined with US-identified HS compared with NAFLD patients who had HS identified by CAP alone in the hemodialysis patients (Table 4). The average bilirubin level, proportion of patients with thrombocytopenia, and LSM value were lower, while the average CAP value and albumin level were higher in NAFLD patients who had CAP combined with US-identified HS compared with NAFLD patients who had HS identified by CAP alone in the control group patients (Table 5).

**Table 4:** Clinical characteristics of hemodialysis patients who were CAP-identified HS with or without US-identified HS.

Variables	CAP ≥ 238 dB/m combined with US-identified HS (N = 62)	CAP ≥ 238 dB/m without US-identified HS (N = 101)	*P* value
Age(yr)	63.9±10.1	64.3±10.5	0.825
Male	27 (43.6%)	45 (44.6%)	0.900
BMI (kg/m^2^)			0.029
<24	29 (47.5%)	64 (63.4%)	
24–30	26 (42.6%)	35(34.7%)	
>30	6 (9.9%)	2 (1.9%)	
DM (%)	27 (43.6%)	36 (35.6%)	0.314
AST (IU/L)	19.6±6.6	19.0±7.0	0.616
ALT (IU/L)	17.4±11.4	15.6±9.4	0.269
Bilirubin (mg/dL)	0.3±0.1	0.3±0.2	0.299
Albumin (g/dL)	4.0±0.3	3.9±0.3	0.255
Fasting sugar (mg/dL)	178.8±111.9	150.7±59.3	0.039
HDL, male< 40, female < 50(mg/dL)	48 (78.7%)	62 (63.3%)	0.041
LDL (mg/dL)	84.7±27.5	91.1±33.0	0.209
Total cholesterol (mg/dL)	163.3±35.3	169.0±38.4	0.351
VLDL(mg/dL)	41.5±18.7	33.1±17.9	0.006
Triglyceride >150 (mg/dL)	42 (67.7%)	51 (51.0%)	0.036
Platelet<150 (1000/μL)	15 (24.2%)	24 (24.0%)	0.978
LSM (kPa)	6.7 (5.3–8.6)	6.3 (4.9–8.2)	0.931
CAP (dB/m)	281 (250–310)	268 (250–286)	0.061

BMI, body mass index; DM, diabetes mellitus; ALT, alanine aminotransferase; AST, aspartate aminotransferase; HDL, High-density lipoprotein; CAP, Controlled Attenuation Parameter; LSM, liver stiffness measurement; LDL, low-density lipoprotein; VLDL, Very-low-density lipoprotein

**Table 5:** Clinical characteristics of the control group patients who were CAP-identified HS with or without US-identified HS.

Variables	CAP ≥ 238 dB/m combined with US-identified HS (N = 160)	CAP ≥ 238 dB/m without US-identified HS (N = 32)	*P* value
Age(yr)	57.7±11.8	58.4±10.3	0.767
Male	81 (50.6%)	17 (53.1%)	0.796
BMI (kg/m^2^)			0.825
<24	33 (21.3%)	7 (23.3%)	
24–30	91 (58.7%)	16 (53.3%)	
>30	31 (20.0%)	7 (23.3%)	
DM (%)	73 (45.6%)	10 (31.25%)	0.134
AST (IU/L)	36.5±25.0	42.2±22.1	0.364
ALT (IU/L)	46.2±32.1	35.7±19.8	0.139
Bilirubin (mg/dL)	0.9±0.7	1.7±2.3	0.015
Albumin (g/dL)	4.7±0.3	4.3±0.4	<0.001
Fasting sugar (mg/dL)	116.4±31.5	107.9±32.0	0.353
HDL, male< 40, female < 50(mg/dL)	28 (20%)	6 (20.7%)	0.933
LDL (mg/dL)	99.5±30.7	101.5±41.5	0.770
Total cholesterol (mg/dL)	182.6±34.9	183.3±44.3	0.919
VLDL(mg/dL)	26.8±12.5	24.4±14.6	0.374
Triglyceride >150 (mg/dL)	45 (32.4%)	7 (24.1%)	0.383
Platelet<150 (1000/μL)	6 (9.8%)	7 (50.0%)	<0.001
LSM (kPa)	5.3 (4.4–7.85)	10 (5.3–25)	<0.001
CAP (dB/m)	293.5 (269–330.5)	269.5 (251–302)	0.004

BMI, body mass index; DM, diabetes mellitus; ALT, alanine aminotransferase; AST, aspartate aminotransferase; HDL, High-density lipoprotein; CAP, Controlled Attenuation Parameter; LSM, liver stiffness measurement; LDL, low-density lipoprotein; VLDL, Very-low-density lipoprotein

## Discussion

To our knowledge, this is the first study using CAP combined with US to survey NAFLD in hemodialysis patients. The main finding of our study was that NAFLD was present in 56% of the hemodialysis patients. Among the hemodialysis patients, the number of diagnoses of HS made by using CAP combined with US was more than twice the number made by using US alone. In contrast, the number of diagnoses of HS made by using CAP combined with US was only slightly higher than the number made by using US alone among the control patients, although the related P value was still significant.

A diagnosis of mild HS made by using US is based on an increased hepatorenal contrast. In most hemodialysis patients, however, the kidneys are atrophied, which makes it difficult to detect if there is any such increased hepatorenal contrast. Therefore, the use of US alone is not feasible for detecting mild HS in ESRD patients with atrophied kidneys.

We used CAP combined with US to determine that more than half of the hemodialysis patients had NAFLD. These patients were at higher risk for developing CVDs, and greater attention should be given to assessments for CVDs in such patients.

As hemodialysis patients are mostly anemic and require transfusions, it was possible that the high CAP values obtained for the hemodialysis patients in this study were caused not only by NAFLD but by iron overloads in their livers. However, the prevalence of iron overload (defined by a serum ferritin level >2000 ng/ml) [25], was just 0.3% among all 343 hemodialysis patients. As such, the high CAP values for this hemodialysis cohort were generally not likely to be related to iron overloads.

Cases of NAFLD missed by US are usually of lower hepatic fat content [26].

The comparison of NAFLD patients who had CAP combined with US-identified HS and NAFLD patients who had HS identified by CAP alone showed that the median CAP values were higher in NAFLD patients who had CAP combined with US-identified HS compared with NAFLD patients who had HS identified by CAP alone in both the hemodialysis and the control group patients, although the P value in the hemodialysis patients was not statistically significant (P = 0.061).

The prevalence of CAP-identified HS among the hemodialysis patients was lower than that among the control population. In addition, the mean BMI in the control population was higher than that among the hemodialysis patients (P<0.001). A previous study enrolled more than 5000 patients on whom CAP was performed, and multivariate analyses showed that the major determinant factor associated with elevated CAP was BMI [27], a finding which is compatible with those of the present study noted above and which could explain the higher prevalence of CAP-identified HS in the control population.

Previous studies have used CAP ≥ 238 dB/m to define HS in hemodialysis patients, and the prevalence of NAFLD in those studies ranged from 52.8–82%. The number of patients in those studies was limited, however, with the largest number of cases in any of the studies being only 94 [20–23].

Furthermore, two of those previous studies enrolled elderly hemodialysis patients, the majority of whom were receiving supportive enteral nutrition [20,21]. In those studies, CAP was negatively correlated with the nutritional parameter (serum albumin), and there was no association between CAP and obesity (as defined by BMI). Additionally, the presence of NAFLD, low serum albumin levels, and high serum C-reactive protein levels were strong predictors of poor outcome in those patients. As a result, the authors of those studies concluded that NAFLD probably interacts with malnutrition, inflammation, and atherosclerosis (MIA) syndrome in hemodialysis patients [20,21]. In contrast, BMI was still an independent factor associated with CAP-identified HS in the hemodialysis patients in our study. The cause of this discrepancy between those previous studies and our study may be our study’s enrollment of much younger patients. The mean ages of the patients in the two previous studies were 75.1±7.1 years and 69.1±12 years, respectively [20,21]. In contrast, the mean age of the hemodialysis patients in this study was 63.2±12.3 years. Elderly patients are at risk of malnutrition [28], and the majority of patients in the two previous studies were receiving supportive enteral nutrition [20,21], indicating that MIA syndrome was present in most of these patients. In contrast, most of the patients in our study were not receiving supportive enteral nutrition. Therefore, we assume that the majority of our patients did not have MIA syndrome.

According to the American Association for the Study of Liver Diseases (AASLD) guidelines, risk factors with an established association with NAFLD include obesity, type 2 DM, dyslipidemia, and metabolic syndrome [24]. In this study, we enrolled patients with normal renal function to serve as a control group in order to investigate whether there are different factors associated with NAFLD in hemodialysis patients versus patients with normal renal function. We found that high BMI and dyslipidemia were factors associated with NAFLD in the hemodialysis patients, while high BMI, high ALT, and low LSM were factors associated with NAFLD in the control group patients. Previous studies have shown that ALT was found to be associated with the clustering of metabolic syndrome components [29,30]. The clinical factors associated with NAFLD in both the hemodialysis and control group patients in this study were compatible with the aforementioned AASLD guidelines [24].

The LSM values of the hemodialysis patients in this study were significantly higher than those of the control subjects, but their CAP values were significantly lower. Furthermore, low LSM was an independent factor associated with US-identified HS in the control group in this study. Recent studies have reported that HS was associated with higher LSM in patients with NAFLD [31,32], which is not compatible with the results of our study. However, as non-alcoholic steatohepatitis (NASH) progress, regression of steatosis, inflammation or ballooning was termed burned-out NASH [33]. Burned-out NASH may explain the negative association between LSM and steatosis in our study.

The current gold standard for evaluating HS is liver biopsy. However, the distribution of fat in the liver is not homogenous. Diffuse HS with focal fatty sparing or focal fatty liver occurs frequently. Relatedly, Ratziu et al. reported an error and misjudgment rate of 24% due to sampling errors in biopsies [34].

Moreover, because liver biopsy is an invasive procedure, it carries a risk of rare but life-threatening complications [35,36]. There are also particular concerns about the risks of performing liver biopsies on ESRD patients on dialysis, with these concerns relating to the fact that such patients have an increased tendency to bleed as a consequence of platelet dysfunction [37]. These limitations have led to the development of non-invasive methods for the assessment of HS.

CAP is non-invasive, quantitative, and easy to perform compared with US, while also having the advantages of being operator- and machine-independent [16]. Furthermore, it can be performed by a technician. As such, and in light of the growing burden of NAFLD, CAP seems to be more suitable than US for the large-scale screening of HS. The main limitation of CAP is in cases of patients with high BMI (BMI>30) [27]. However, among the hemodialysis patients in this study, only 2.4% had BMI >30. Therefore, CAP seems to be an ideal tool with which to screen for HS in a large cohort of hemodialysis patients.

The US evaluation of steatosis is mainly qualitative [38]. The qualitative grading is conveniently classified as mild, moderate or severe [19]. In contrast, the CAP evaluation of steatosis is mainly quantitative. It is not surprising that the agreement between the two examinations is not good in both the hemodialysis and the control group patients, and significant proportion of patients had discordant results between CAP and US in our study.

There are limitations to this study. Firstly, there was a lack of data regarding the etiology of ESRD, as well as the frequency, duration, and dose of dialysis, for the hemodialysis patients. Secondly, we also did not have liver biopsy data for the patients, even though liver biopsy is the gold standard for evaluating HS. Furthermore, the cutoff value of CAP used for diagnosing HS was obtained from non-uremic populations [16].

The present study also has several strengths. Using CAP combined with US to survey NAFLD in hemodialysis patients, we found that more than half of the hemodialysis patients had NAFLD. These patients were at higher risk for developing CVDs, and greater attention should be given to assessments for CVDs in such patients. Furthermore, US is not suitable for diagnosing steatosis in ESRD patients due to kidney atrophy. Only 26.5% of the hemodialysis patients in this study had US-identified HS, which was significantly lower than the percentage with CAP combined with US-identified HS. In contrast, the number of diagnoses of HS made by using CAP combined with US was only slightly higher than the number made with US alone in the control group patients. These results suggest that, although US is widely available, it may severely underdiagnose HS in ESRD patients.

## Supporting information

S1 Dataset The raw data of the hemodialysis patients. (XLS)

S2 Dataset The raw data of the control group patients. (XLSX)

S1 Supplementary Tables (DOCX)

## References

[1] Locatelli F, Pozzoni P, Tentori F, del Vecchio L. Epidemiology of cardiovascular risk in patients with chronic kidney disease. Nephrol Dial Transplant. 2003;18 Suppl 7:vii2–9. 12953023 10.1093/ndt/gfg1072

[2] Hamad AA, Khalil AA, Connolly V, Ahmed MH. Relationship between non-alcoholic fatty liver disease and kidney function: a communication between two organs that needs further exploration. Arab J Gastroenterol. 2012; 13:161–5. doi: 10.1016/j.ajg.2012.06.010 23432982 10.1016/j.ajg.2012.06.010

[3] de Jager DJ, Grootendorst DC, Jager KJ, van Dijk PC, Tomas LM, Ansell D, et al Cardiovascular and noncardiovascular mortality among patients starting dialysis. JAMA. 2009; 302:1782–9. doi: 10.1001/jama.2009.1488 19861670 10.1001/jama.2009.1488

[4] Sniderman AD, Solhpour A, Alam A, Williams K, Sloand JA. Cardiovascular death in dialysis patients: lessons we can learn from AURORA. Clin J Am Soc Nephrol. 2010;5:335–40. doi: 10.2215/CJN.06300909 20056762 10.2215/CJN.06300909

[5] Vanholder R, Massy Z, Argiles A, Spasovski G, Verbeke F, Lameire N; European Uremic Toxin Work Group. European Uremic Toxin Work Group. Chronic kidney disease as cause of cardiovascular morbidity and mortality. Nephrol Dial Transplant. 2005; 20:1048–56. doi: 10.1093/ndt/gfh813 15814534 10.1093/ndt/gfh813

[6] Lai YC, Cheng BC, Hwang JC, Lee YT, Chiu CH, Kuo LC, et al Association of fatty liver disease with nonfatal cardiovascular events in patients undergoing maintenance hemodialysis. Nephron Clin Pract. 2013;124:218–23. doi: 10.1159/000357952 24503573 10.1159/000357952

[7] Longenecker JC, Coresh J, Powe NR, Levey AS, Fink NE, Martin A, et al Traditional cardiovascular disease risk factors in dialysis patients compared with the general population: the CHOICE Study. J Am Soc Nephrol. 2002;13:1918–27. 12089389 10.1097/01.asn.0000019641.41496.1e

[8] D'Adamo E, Cali AM, Weiss R, Santoro N, Pierpont B, Northrup V, et al Central role of fatty liver in the pathogenesis of insulin resistance in obese adolescents. Diabetes Care. 2010;33:1817–22. doi: 10.2337/dc10-0284 20668154 10.2337/dc10-0284 PMC2909068

[9] Kotronen A, Yki-Järvinen H. Fatty liver: a novel component of the metabolic syndrome. Arterioscler Thromb Vasc Biol. 2008;28:27–38. doi: 10.1161/ATVBAHA.107.147538 17690317 10.1161/ATVBAHA.107.147538

[10] Kotronen A, Velagapudi VR, Yetukuri L, Westerbacka J, Bergholm R, Ekroos K, et al Serum saturated fatty acids containing triacylglycerols are better markers of insulin resistance than total serum triacylglycerol concentrations. Diabetologia. 2009;52:684–90. doi: 10.1007/s00125-009-1282-2 19214471 10.1007/s00125-009-1282-2

[11] Targher G, Day CP, Bonora E. Risk of cardiovascular disease in patients with nonalcoholic fatty liver disease. N Engl J Med. 2010;363:1341–50. doi: 10.1056/NEJMra0912063 20879883 10.1056/NEJMra0912063

[12] Pacifico L, Nobili V, Anania C, Verdecchia P, Chiesa C. Pediatric nonalcoholic fatty liver disease, metabolic syndrome and cardiovascular risk. World J Gastroenterol. 2011;17:3082–91. doi: 10.3748/wjg.v17.i26.3082 21912450 10.3748/wjg.v17.i26.3082 PMC3158407

[13] Schwimmer JB, Pardee PE, Lavine JE, Blumkin AK, Cook S. Cardiovascular risk factors and the metabolic syndrome in pediatric nonalcoholic fatty liver disease. Circulation. 2008; 118:277–83. doi: 10.1161/CIRCULATIONAHA.107.739920 18591439 10.1161/CIRCULATIONAHA.107.739920 PMC2996820

[14] Anstee QM, Targher G, Day CP. Progression of NAFLD to diabetes mellitus, cardiovascular disease or cirrhosis. Nat Rev Gastroenterol Hepatol. 2013; 10:330–44. doi: 10.1038/nrgastro.2013.41 23507799 10.1038/nrgastro.2013.41

[15] Mikolasevic I, Racki S, Zaputovic L, Lukenda V, Milic S, Orlic L. Nonalcoholic fatty liver disease (NAFLD): a new risk factor for adverse cardiovascular events in dialysis patients. Med Hypotheses. 2014; 82:205–8. doi: 10.1016/j.mehy.2013.11.039 24365277 10.1016/j.mehy.2013.11.039

[16] Sasso M, Beaugrand M, de Ledinghen V, Douvin C, Marcellin P, Poupon R, et al Controlled attenuation parameter (CAP): a novel VCTE™ guided ultrasonic attenuation measurement for the evaluation of hepatic steatosis: preliminary study and validation in a cohort of patients with chronic liver disease from various causes. Ultrasound Med Biol. 2010; 36:1825–35. doi: 10.1016/j.ultrasmedbio.2010.07.005 20870345 10.1016/j.ultrasmedbio.2010.07.005

[17] Shi KQ, Tang JZ, Zhu XL, Ying L, Li DW, Gao J, et al Controlled attenuation parameter for the detection of steatosis severity in chronic liver disease: a meta-analysis of diagnostic accuracy. J Gastroenterol Hepatol. 2014; 29:1149–58. doi: 10.1111/jgh.12519 24476011 10.1111/jgh.12519

[18] Hamaguchi M, Kojima T, Itoh Y, Harano Y, Fujii K, Nakajima T, et al The severity of ultrasonographic findings in nonalcoholic fatty liver disease reflects the metabolic syndrome and visceral fat accumulation. Am J Gastroenterol 2007;102:2708–2715. doi: 10.1111/j.1572-0241.2007.01526.x 17894848 10.1111/j.1572-0241.2007.01526.x

[19] Dasarathy S, Dasarathy J, Khiyami A, Joseph R, Lopez R, McCullough AJ. Validity of real time ultrasound in the diagnosis of hepatic steatosis: a prospective study. J Hepatol 2009;51:1061–1067. doi: 10.1016/j.jhep.2009.09.001 19846234 10.1016/j.jhep.2009.09.001 PMC6136148

[20] Mikolasevic I, Lukenda V, Racki S, Milic S, Sladoje-Martinovic B, Orlic L. Nonalcoholic fatty liver disease (NAFLD)—a new factor that interplays between inflammation, malnutrition, and atherosclerosis in elderly hemodialysis patients. Clin Interv Aging. 2014;9:1295–303. doi: 10.2147/CIA.S65382 25143715 10.2147/CIA.S65382 PMC4132229

[21] Mikolasevic I, Stimac D, Racki S, Zaputovic L, Devcic B, Jelic I, et al Relationship between non-alcoholic fatty liver disease and MIA syndrome. Hemodial Int. 2015;19:472–81. doi: 10.1111/hdi.12280 25688578 10.1111/hdi.12280

[22] Mikolasevic I, Orlic L, Zaputovic L, Racki S, Cubranic Z, Anic K, et al Usefulness of liver test and controlled attenuation parameter in detection of nonalcoholic fatty liver disease in patients with chronic renal failure and coronary heart disease. Wien Klin Wochenschr. 2015;127:451–8. doi: 10.1007/s00508-015-0757-z 25854911 10.1007/s00508-015-0757-z

[23] Mikolasevic I, Orlic L, Milic S, Zaputovic L, Lukenda V, Racki S. Non-alcoholic fatty liver disease proven by transient elastography in hemodialysis patients: is it a new risk factor for adverse cardiovascular events? Blood Purif. 2014;37:259–65. doi: 10.1159/000360270 24993140 10.1159/000360270

[24] Chalasani N, Younossi Z, Lavine JE, Diehl AM, Brunt EM, Cusi K, et al The diagnosis and management of non-alcoholic fatty liver disease: practice Guideline by the American Association for the Study of Liver Diseases, American College of Gastroenterology, and the American Gastroenterological Association. Hepatology. 2012; 55:2005–23. doi: 10.1002/hep.25762 22488764 10.1002/hep.25762

[25] Chen CH, Shu KH, Yang Y. Long-term effects of an oral iron chelator, deferasirox, in hemodialysis patients with iron overload. Hematology. 2015; 20:304–10. doi: 10.1179/1607845414Y.0000000199 25200910 10.1179/1607845414Y.0000000199

[26] Yilmaz Y, Ergelen R, Akin H, Imeryuz N. Noninvasive detection of hepatic steatosis in patients without ultrasonographic evidence of fatty liver using the controlled attenuation parameter evaluated with transient elastography. Eur J Gastroenterol Hepatol. 2013; 25:1330–4. doi: 10.1097/MEG.0b013e3283623a16 23660937 10.1097/MEG.0b013e3283623a16

[27] de Lédinghen V, Vergniol J, Capdepont M, Chermak F, Hiriart JB, Cassinotto C, et al Controlled attenuation parameter (CAP) for the diagnosis of steatosis: a prospective study of 5323 examinations. J Hepatol. 2014;60:1026–31. doi: 10.1016/j.jhep.2013.12.018 24378529 10.1016/j.jhep.2013.12.018

[28] Stenvinkel P, Heimbürger O, Paultre F, Diczfalusy U, Wang T, Berglund L, et al Strong association between malnutrition, inflammation, and atherosclerosis in chronic renal failure. Kidney Int. 1999;55:1899–911. doi: 10.1046/j.1523-1755.1999.00422.x 10231453 10.1046/j.1523-1755.1999.00422.x

[29] Lee K, Yang JH. Which liver enzymes are better indicators of metabolic syndrome in adolescents: the Fifth Korea National Health and Nutrition Examination Survey, 2010. Metab Syndr Relat Disord. 2013;11:229–35. doi: 10.1089/met.2012.0153 23451816 10.1089/met.2012.0153

[30] Hanley AJ, Williams K, Festa A, Wagenknecht LE, D'Agostino RB Jr, Haffner SM. Liver markers and development of the metabolic syndrome: the insulin resistance atherosclerosis study. Diabetes. 2005;54:3140–7. 16249437 10.2337/diabetes.54.11.3140

[31] Petta S, Maida M, Macaluso FS, Di Marco V, Cammà C, Cabibi D, et al The severity of steatosis influences liver stiffness measurement in patients with nonalcoholic fatty liver disease. Hepatology. 2015;62:1101–10. doi: 10.1002/hep.27844 25991038 10.1002/hep.27844

[32] Petta S, Wai-Sun Wong V, Cammà C, Hiriart JB, Wong GL, Marra F, et al Improved Noninvasive prediction of Liver Fibrosis by Liver Stiffness Measurement in Patients with Nonalcoholic Fatty Liver Disease Accounting for Controlled Attenuation Parameter Values. Hepatology. 2016 9 17. 10.1002/hep.28843 27639088

[33] European Association for the Study of the Liver (EASL).; European Association for the Study of Diabetes (EASD).; European Association for the Study of Obesity (EASO). EASL-EASD-EASO Clinical Practice Guidelines for the management of non-alcoholic fatty liver disease.J Hepatol. 2016;64:1388–402. doi: 10.1016/j.jhep.2015.11.004 27062661 10.1016/j.jhep.2015.11.004

[34] Ratziu V, Charlotte F, Heurtier A, Gombert S, Giral P, Bruckert E, et al Sampling variability of liver biopsy in nonalcoholic fatty liver disease. Gastroenterology. 2005; 128:1898–906. 15940625 10.1053/j.gastro.2005.03.084

[35] Bravo AA, Sheth SG, Chopra S. Liver biopsy. N Engl J Med 2001;344: 495–500. doi: 10.1056/NEJM200102153440706 11172192 10.1056/NEJM200102153440706

[36] Piccinino F, Sagnelli E, Pasquale G, Giusti G. Complications following percutaneous liver biopsy. A multicentre retrospective study on 68,276 biopsies. J Hepatol 1986;2:165–173. 3958472 10.1016/s0168-8278(86)80075-7

[37] Brophy DF, Martin EJ, Carr SL, Kirschbaum B, Carr ME Jr. The effect of uremia on platelet contractile force, clot elastic modulus and bleeding time in hemodialysis patients. Thromb Res 2007;119:723–729. doi: 10.1016/j.thromres.2006.02.013 16793120 10.1016/j.thromres.2006.02.013

[38] Chen CL, Cheng YF, Yu CY, Ou HY, Tsang LL, Huang TL, et al Living donor liver transplantation: the Asian perspective. Transplantation 2014;97 Suppl 8:S3. 10.1097/TP.0000000000000060 24849827

