# Supplementary material for: Using controlled attenuation parameter combined with ultrasound to survey non-alcoholic fatty liver disease in hemodialysis patients: A prospective cohort study
Source: PLoS One. 2017 Apr 20;12(4):e0176027. doi: 10.1371/journal.pone.0176027 (PMC5398606; doi:10.1371/journal.pone.0176027)
Supplement: S1 Supplementary Tables — (DOCX) [file pone.0176027.s003.docx]

Table A. Clinical characteristics of patients with CAP-identified NAFLD, but not US-identified NAFLD

| Variables | Hemodialysis (N=101) | control(N=32) | *P* value |
| --- | --- | --- | --- |
| Age(yr) | 64.3±10.5 | 58.4±10.3 | 0.007 |
| Male | 45 (44.6%) | 17 (53.1%) | 0.397 |
| BMI (kg/m^2^) |  |  | <0.001 |
| <24 | 64 (63.4%) | 7 (23.3%) |  |
| 24-30 | 35 (34.6%) | 16 (53.4%) |  |
| >30 | 2 (2.0%) | 7 (23.3%) |  |
| DM (%) | 36 (35.6%) | 10 (31.3%) | 0.649 |
| AST (IU/L) | 19.0±7.0 | 42.2±22.1 | <0.001 |
| ALT (IU/L) | 15.6±9.4 | 35.7±19.8 | <0.001 |
| Bilirubin (mg/dL) | 0.3±0.1 | 1.7±2.3 | <0.001 |
| Albumin (g/dL) | 3.9±0.3 | 4.3±0.4 | <0.001 |
| Fasting sugar (mg/dL) | 150.9±59.3 | 107.9±32.0 | 0.007 |
| HDL, male< 40, female < 50(mg/dL) | 62 (63.3%) | 6 (20.7%) | <0.001 |
| LDL (mg/dL) | 91.1±33.0 | 101.5±41.5 | 0.171 |
| Total cholesterol (mg/dL) | 169.0±38.4 | 183.3±44.3 | 0.089 |
| VLDL(mg/dL) | 33.1±17.9 | 24.4±14.6 | 0.019 |
| Triglyceride >150 (mg/dL) | 51 (51.0%) | 7 (24.1%) | 0.010 |
| Platelet<150 (1000/μL) | 24 (24.0%) | 7 (50.0%) | 0.041 |
| LSM (kPa) | 6.3 (4.9-8.2) | 10 (5.3-25) | <0.001 |
| CAP (dB/m) | 268 (250-286) | 269.5 (251-302) | 0.481 |

data are shown as mean±SD, but LSM, and CAP are shown as median (interquantile).

NAFLD, non-alcoholic fatty liver disease; BMI, body mass index; DM, diabetes mellitus; ALT, alanine aminotransferase; AST, aspartate aminotransferase; HDL, High-density lipoprotein; CAP, Controlled Attenuation Parameter; LSM, liver stiffness measurement; LDL, low-density lipoprotein; VLDL, Very-low-density lipoprotein;

Table B. Clinical characteristics of patients with discordant results between CAP and US-identified NAFLD in hemodialysis patients

| Variables | CAP≥238 dB/m without US-identified HS (N=101) | US-identified HS with CAP< 238 dB/m (N=29) | *P* value |
| --- | --- | --- | --- |
| Age(yr) | 64.3±10.5 | 64.8±9.7 | 0.793 |
| Male | 45 (44.6%) | 14 (48.3%) | 0.723 |
| BMI (kg/m^2^) |  |  | 0.894 |
| <24 | 64 (63.4%) | 20 (69.0%) |  |
| 24-30 | 35 (34.7%) | 9 (31.0%) |  |
| >30 | 2 (1.9%) | 0 (0.0%) |  |
| DM (%) | 36 (35.6%) | 7 (24.1%) | 0.246 |
| AST (IU/L) | 19.0±7.0 | 20.4±9.3 | 0.406 |
| ALT (IU/L) | 15.6±9.4 | 19.3±16.9 | 0.118 |
| Bilirubin (mg/dL) | 0.3±0.1 | 0.4±0.2 | 0.131 |
| Albumin (g/dL) | 3.9±0.3 | 3.9±0.3 | 0.696 |
| Fasting sugar (mg/dL) | 150.9±59.3 | 150.0±64.8 | 0.945 |
| HDL, male< 40, female < 50(mg/dL) | 62 (63.3%) | 14 (50.0%) | 0.206 |
| LDL (mg/dL) | 91.1±33.0 | 91.6±26.7 | 0.945 |
| Total cholesterol (mg/dL) | 169.0±38.4 | 174.1±49.1 | 0.558 |
| VLDL(mg/dL) | 33.1±17.9 | 28.9±16.9 | 0.264 |
| Triglyceride >150 (mg/dL) | 51 (51.0%) | 10 (35.7%) | 0.152 |
| Platelet<150 (1000/μL) | 24 (24.0%) | 9 (32.1%) | 0.384 |
| LSM (kPa) | 6.3 (4.9-8.2) | 5.2 (4-6.6) | 0.030 |
| CAP (dB/m) | 268 (250-286) | 203 (184-224) | <0.001 |

data are shown as mean±SD, but LSM, and CAP are shown as median (interquantile).

NAFLD, non-alcoholic fatty liver disease; BMI, body mass index; DM, diabetes mellitus; ALT, alanine aminotransferase; AST, aspartate aminotransferase; HDL, High-density lipoprotein; CAP, Controlled Attenuation Parameter; LSM, liver stiffness measurement; LDL, low-density lipoprotein; VLDL, Very-low-density lipoprotein;

Table C. Clinical characteristics of patients with discordant results between CAP and US-identified NAFLD in control group patients

| Variables | CAP≥238 dB/m without US-identified HS (N=32) | US-identified HS with CAP< 238 dB/m (N=20) | *P* value |
| --- | --- | --- | --- |
| Age(yr) | 58.4±10.3 | 60.0±7.1 | 0.539 |
| Male | 17 (53.1%) | 6 (30.0%) | 0.102 |
| BMI (kg/m^2^) |  |  | 0.044 |
| <24 | 7 (23.3%) | 8 (47.1%) |  |
| 24-30 | 16 (53.3%) | 9 (52.9%) |  |
| >30 | 7 (23.3%) | 0 (0.0%) |  |
| DM (%) | 10 (31.25%) | 10 (50.0%) | 0.176 |
| AST (IU/L) | 42.2±22.1 | 28.2±19.5 | 0.117 |
| ALT (IU/L) | 35.7±19.8 | 34.0±41.1 | 0.871 |
| Bilirubin (mg/dL) | 1.7±2.3 | 0.6±0.2 | 0.200 |
| Albumin (g/dL) | 4.3±0.4 | 4.7±0.1 | 0.082 |
| Fasting sugar (mg/dL) | 107.9±32.0 | 104.9±16.8 | 0.815 |
| HDL, male< 40, female < 50(mg/dL) | 6 (20.7%) | 2 (11.8%) | 0.441 |
| LDL (mg/dL) | 101.5±41.5 | 99.0±24.2 | 0.834 |
| Total cholesterol (mg/dL) | 183.3±44.3 | 171.1±34.8 | 0.333 |
| VLDL(mg/dL) | 24.4±14.6 | 22.3±7.7 | 0.603 |
| Triglyceride >150 (mg/dL) | 7 (24.1%) | 2 (11.8%) | 0.307 |
| Platelet<150 (1000/μL) | 7 (50%) | 1 (14.3%) | 0.112 |
| LSM (kPa) | 10 (5.3-25) | 4.4 (3.45-4.7) | 0.002 |
| CAP (dB/m) | 269.5 (251-302) | 225.5 (215.5-232.5) | <0.001 |

data are shown as mean±SD, but LSM, and CAP are shown as median (interquantile).

NAFLD, non-alcoholic fatty liver disease; BMI, body mass index; DM, diabetes mellitus; ALT, alanine aminotransferase; AST, aspartate aminotransferase; HDL, High-density lipoprotein; CAP, Controlled Attenuation Parameter; LSM, liver stiffness measurement; LDL, low-density lipoprotein; VLDL, Very-low-density lipoprotein;

Table D. Factors associated with CAP ≥ 248 dB/m vs. CAP <248 dB/m (logistic regression).

| Variables |  | Hemodialysis | | | | |  | Control | | | | |
| --- | --- | --- | --- | --- | --- | --- | --- | --- | --- | --- | --- | --- |
|  |  | Univariate | |  | Multivariate | |  | Univariate | |  | Multivariate | |
|  |  | OR (95% CI) | *P* |  | OR | *P* |  | OR (95% CI) | *P* |  | OR | *P* |
| Age |  | 1.01 (0.99-1.03) | 0.332 |  | - |  |  | 1.01 (0.99-1.03) | 0.475 |  | - |  |
| Male |  | 1.01 (0.65-1.57) | 0.955 |  | - |  |  | 1.05 (0.61-1.79) | 0.866 |  | - |  |
| BMI (kg/m^2^) |  | 1.40 (1.28-1.52) | <0.001 |  | 1.30 (1.19-1.43) | <0.001 |  | 1.32 (1.20-1.45) | <0.001 |  | 1.28 (1.16-1.41) | <0.001 |
| DM |  | 2.13 (1.33-3.41) | 0.002 |  | 1.22 (0.64-2.30) | 0.551 |  | 2.15 (1.20-3.86) | 0.010 |  | 2.06 (1.03-4.13) | 0.042 |
| AST (IU/L) |  | 0.99 (0.97-1.02) | 0.490 |  | - |  |  | 1.01 (0.99-1.03) | 0.198 |  | - |  |
| ALT(IU/L) |  | 1.00 (0.98-1.02) | 0.820 |  | - |  |  | 1.01 (1.00-1.03) | 0.087 |  | - |  |
| Fasting sugar (mg/dL) |  | 1.01 (1.00-1.01) | <0.001 |  | 1.004 (0.99-1.01) | 0.167 |  | 1.01 (1.00-1.03) | 0.136 |  | - |  |
| HDL, male< 40, female < 50(mg/dL) |  | 3.92 (2.43-6.31) | <0.001 |  | 1.97 (1.13-3.44) | 0.017 |  | 2.32 (0.91-5.87) | 0.076 |  | - |  |
| LDL(mg/dL) |  | 1.00 (0.99-1.00) | 0.380 |  | - |  |  | 1.00 (0.99-1.01) | 0.511 |  | - |  |
| Total cholesterol(mg/dL) |  | 1.00 (1.00-1.01) | 0.585 |  | - |  |  | 1.00 (0.99-1.01) | 0.566 |  | - |  |
| Triglyceride >150(mg/dL) |  | 4.59 (2.86-7.36) | <0.001 |  | 2.30 (1.32-1.00) | 0.003 |  | 5.40 (2.03-14.33) | 0.001 |  | 4.36 (1.44-13.22) | 0.009 |
| Platelet(1000/μL) |  | 0.76 (0.46-1.25) | 0.283 |  | - |  |  | 0.45 (0.17-1.18) | 0.104 |  | - |  |
| LSM (kPa) |  | 1.03 (0.97-1.09) | 0.296 |  | - |  |  | 1.00 (0.98-1.03) | 0.728 |  | - |  |

BMI, body mass index; DM, diabetes mellitus; ALT, alanine aminotransferase; AST, aspartate aminotransferase; HDL, High-density lipoprotein; CAP, Controlled Attenuation Parameter; LSM, liver stiffness measurement; LDL, low-density lipoprotein; VLDL, Very-low-density lipoprotein;

Table E. The prevalence of NAFLD in DM/non-DM, obese/non-obese, high/low triglyceride patients.

|  |  | Hemodialysis |  |  | Control |  |
| --- | --- | --- | --- | --- | --- | --- |
| Variables | Total cases | NAFLD cases | Prevalence | Total cases | NAFLD cases | Prevalence |
| DM |  |  |  |  |  |  |
| No | 238 | 122 | 51.3% | 154 | 119 | 77.3% |
| Yes | 105 | 70 | 66.7% | 98 | 93 | 94.9% |
| BMI (kg/m^2^) |  |  |  |  |  |  |
| <24 | 246 | 113 | 45.9% | 73 | 48 | 65.8% |
| ≥24 | 95 | 78 | 82.1% | 168 | 154 | 91.7% |
| Triglyceride >150 (mg/dL) |  |  |  |  |  |  |
| No | 213 | 87 | 40.8% | 156 | 131 | 84.0% |
| Yes | 126 | 103 | 81.7% | 56 | 54 | 96.4% |

NAFLD, non-alcoholic fatty liver disease; BMI, body mass index; DM, diabetes mellitus;
